# Supplementary material for: Characteristics of the right ventricle in left ventricular noncompaction with reduced ejection fraction in the light of dilated cardiomyopathy
Source: PLoS One. 2023 Sep 25;18(9):e0290981. doi: 10.1371/journal.pone.0290981 (PMC10519585; doi:10.1371/journal.pone.0290981)
Supplement: S1 Table — DCM: dilated cardiomyopathy, LVNC-R: left ventricular noncompaction with reduced LV function, LVNC-N: left ventricular noncompaction with good LV function, ECG: electrocardiography. The bold values indicate statistical significances (p<0.05). (DOCX) [file pone.0290981.s001.docx]

Supporting Information

**Table S1** - Clinical features of the studied patient groups

| Clinical symptoms (%) | DCM | LVNC-R | LVNC-N | p (LVNC-R vs. DCM) | p (LVNC-R vs. LVNC-N) | P (DCM vs. LVNC-N) |
| --- | --- | --- | --- | --- | --- | --- |
| Heart failure | 64.5 | 70.5 | 0.0 | 0.587 | **0.0001** | **0.0001** |
| Palpitation | 35.5 | 18.2 | 29.5 | 0.090 | 0.211 | 0.587 |
| Documented arrhythmia | 58.1 | 52.3 | 27.3 | 0.620 | **0.017** | **0.007** |
| Syncope | 19.4 | 18.2 | 11.4 | 0.898 | 0.367 | 0.335 |
| ECG abnormalities | 64.5 | 68.2 | 13.6 | 0.740 | **0.0001** | **0.0001** |
| Thromboembolic event | 19.4 | 29.6 | 4.5 | 0.318 | **0.002** | **0.041** |
| Sudden cardiac death | 6.5 | 4.5 | 0.0 | 0.718 | 0.153 | 0.088 |
| Positive family history | 38.7 | 20.5 | 36.4 | 0.083 | 0.098 | 0.836 |

DCM: dilated cardiomyopathy, LVNC-R: left ventricular noncompaction with reduced LV function, LVNC-N: left ventricular noncompaction with good LV function, ECG: electrocardiography

The bold values indicate statistical significances (p<0.05).
