# Supplementary material for: Characteristics of the right ventricle in left ventricular noncompaction with reduced ejection fraction in the light of dilated cardiomyopathy
Source: PLoS One. 2023 Sep 25;18(9):e0290981. doi: 10.1371/journal.pone.0290981 (PMC10519585; doi:10.1371/journal.pone.0290981)
Supplement: S2 Table — Exclusion criteria: other technical reasons: implanted devices, arrhythmic or respiratory artifacts. comorbidities: ischemic, valvular, or congenital heart diseases, coexisting cardiomyopathies; hypertension, diabetes mellitus, endocrine disorders, chronic kidney or systemic diseases. intense sports activity: >6 hours/week. LVNC: left ventricular noncompaction, DCM: dilated cardiomyopathy, LV: left ventricle, LVNC-R: left ventricular noncompaction with reduced LV function, LVNC-N: left ventricular noncompaction with good LV function. (DOCX) [file pone.0290981.s002.docx]

Supporting Information


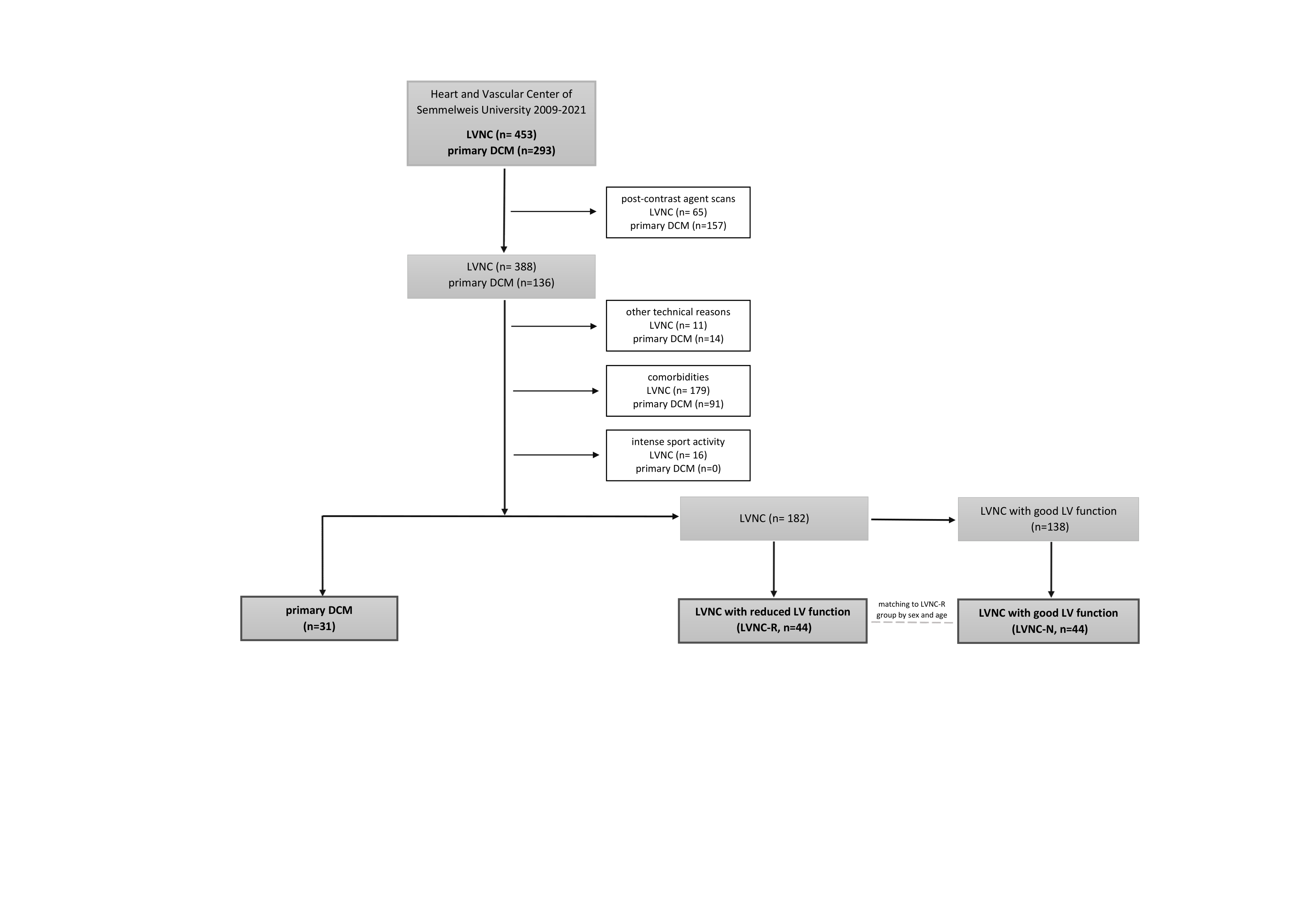
**Table S2** - Flowchart of the enrollment process

Exclusion criteria: *other technical reasons*: implanted devices, arrhythmic or respiratory artifacts

*comorbidities*: ischemic, valvular, or congenital heart diseases, coexisting cardiomyopathies;

hypertension, diabetes mellitus, endocrine disorders, chronic kidney or systemic diseases

*intense sports activity*: >6 hours/week

LVNC: left ventricular noncompaction, DCM: dilated cardiomyopathy, LV: left ventricle, LVNC-R: left ventricular noncompaction with reduced LV function, LVNC-N: left ventricular noncompaction with good LV function
