# Supplementary material for: Characteristics of the right ventricle in left ventricular noncompaction with reduced ejection fraction in the light of dilated cardiomyopathy
Source: PLoS One. 2023 Sep 25;18(9):e0290981. doi: 10.1371/journal.pone.0290981 (PMC10519585; doi:10.1371/journal.pone.0290981)
Supplement: S3 Table — ICC: intraclass correlation coeffitients, RV-EDVi: right ventricular end-diastolic volume index, RV-ESVi: right ventricular end-systolic volume index, RV-SVi: right ventricular stroke volume index, RV-EF: right ventricular ejection fraction, RV-TotalMassi: right ventricular end-diastolic total myocardial mass index, RV-TMi: right ventricular end-diastolic trabecular and papillary muscle mass index, RV-GLS: right ventricular global longitudinal strain, RV-FWS: right ventricular free-wall strain, RV-SS: right ventricular septal strain. (DOCX) [file pone.0290981.s003.docx]

Supporting Information

**Table S3** - Interobserver variability: the intraclass correlation coefficient was interpreted as less than 0.4, between 0.4 and 0.75, and greater than 0.75 indicated poor, fair to good and excellent interobserver agreement, respectively.

|  | ICC |
| --- | --- |
| RV-EDVi (ml/m^2^) | 0.988 (0.966-0.996) |
| RV-ESVi (ml/m^2^) | 0.996 (0.987-0.998) |
| RV-SVi (ml/m^2^) | 0.931 (0.812-0.976) |
| RV-EF (%) | 0.972 (0.919-0.990) |
| RV-TotalMassi (g/m^2^) | 0.989 (0.968-0.996) |
| RV-TMi (g/m^2^) | 0.965 (0.728-0.991) |
| RV-GLS (%) | 0.900 (0.735-0.965) |
| RV-FWS (%) | 0.919 (0.776-0-972) |
| RV-SS (%) | 0.856 (0.623-0.950) |

ICC: intraclass correlation coeffitients, RV-EDVi: right ventricular end-diastolic volume index, RV-ESVi: right ventricular end-systolic volume index, RV-SVi: right ventricular stroke volume index, RV-EF: right ventricular ejection fraction, RV-TotalMassi: right ventricular end-diastolic total myocardial mass index, RV-TMi: right ventricular end-diastolic trabecular and papillary muscle mass index, RV-GLS: right ventricular global longitudinal strain, RV-FWS: right ventricular free-wall strain, RV-SS: right ventricular septal strain
