# Supplementary material for: Characteristics of the right ventricle in left ventricular noncompaction with reduced ejection fraction in the light of dilated cardiomyopathy
Source: PLoS One. 2023 Sep 25;18(9):e0290981. doi: 10.1371/journal.pone.0290981 (PMC10519585; doi:10.1371/journal.pone.0290981)
Supplement: S4 Table — DCM-NT: dilated cardiomyopathy with normal right ventricular trabeculation, DCM-HT: dilated cardiomyopathy with right ventricular hypertrabeculation, LVNC-R-NT: left ventricular noncompaction with reduced left ventricular function and normal right ventricular trabeculation, LVNC-R-HT: left ventricular noncompaction with reduced left ventricular function and right ventricular hypertrabeculation, LVNC-N-NT: left ventricular noncompaction with good left ventricular function and normal right ventricular trabeculation, LVNC-N-HT: left ventricular noncompaction with good left ventricular function and right ventricular hypertrabeculation. RV-EDVi: right ventricular end-diastolic volume index, RV-ESVi: right ventricular end-systolic volume index, RV-SVi: right ventricular stroke volume index, RV-EF: right ventricular ejection fraction, RV-TMi: right ventricular end-diastolic trabecular and papillary muscle mass index, RV-CMi: right ventricular end-diastolic compact myocardial mass index, RV-GLS: right ventricular global longitudinal strain, RV-FWS: right ventricular free-wall strain, RV-SS: right ventricular septal strain. The bold values indicate statistical significances (p<0.05). (DOCX) [file pone.0290981.s004.docx]

Supporting Information

**Table S4** – Comparison of the subgroups with normal RV trabeculation (NT) and with RV hypertrabeculation (HT) within the groups

|  | DCM | | | LVNC-R | | | LVNC-N | | |
| --- | --- | --- | --- | --- | --- | --- | --- | --- | --- |
|  | **NT** | **HT** | **p** | **NT** | **HT** | **p** | **NT** | **HT** | **p** |
| RV-EDVi (ml/m^2^) | 53.4±13.6 | 69.0±17.4 | **0.009** | 51.2±11.7 | 70.0±14.9 | **0.0001** | 68.1±14.2 | 73.7±16.0 | 0.218 |
| RV-ESVi (ml/m^2^) | 19.1±6.9 | 32.1±11.9 | **0.002** | 17.7±5.5 | 32.7±11.4 | **0.0001** | 25.6±7.9 | 28.6±6.4 | 0.180 |
| RV-SVi (ml/m^2^) | 34.4±10.2 | 33.6±8.4 | 0.820 | 33.6±8.3 | 35.8±10.6 | 0.454 | 42.9±7.2 | 44.2±8.9 | 0.584 |
| RV-EF (%) | 64.1±10.5 | 51.1±14.5 | **0.015** | 65.9±7.0 | 52.1±13.0 | **0.0001** | 63.9±6.8 | 59.2±7.6 | 0.074 |
| RV-TMi (g/m^2^) | 18.2±3.5 | 26.8±5.7 | **0.0001** | 17.2±2.3 | 30.0±8.3 | **0.0001** | 18.5±4.5 | 26.7±6.3 | **0.0001** |
| RV-CMi (g/m^2^) | 12.6±3.6 | 15.8±4.7 | **0.04** | 12.6±2.1 | 16.5±4.1 | **0.0001** | 13.4±3.2 | 16.1±4.8 | **0.037** |
| RV-GLS (%) | -19.5±4.6 | -20.2±6.8 | 0.722 | -22.8±7.5 | -17.5±5.9 | **0.014** | -24.9±4.7 | -23.4±3.2 | 0.229 |
| RV-FWS (%) | -22.3±7.9 | -20.9±10.5 | 0.666 | -26.5±8.5 | -19.8±8.8 | **0.013** | -28.8±5.8 | -27.6±4.0 | 0.442 |
| RV-SS (%) | -12.3±7.1 | -13.7±7.8 | 0.619 | -13.3±5.8 | -9.4±6.4 | **0.040** | -14.8±5.2 | -13.4±4.4 | 0.354 |

DCM-NT: dilated cardiomyopathy with normal right ventricular trabeculation, DCM-HT: dilated cardiomyopathy with right ventricular hypertrabeculation, LVNC-R-NT: left ventricular noncompaction with reduced left ventricular function and normal right ventricular trabeculation, LVNC-R-HT: left ventricular noncompaction with reduced left ventricular function and right ventricular hypertrabeculation, LVNC-N-NT: left ventricular noncompaction with good left ventricular function and normal right ventricular trabeculation, LVNC-N-HT: left ventricular noncompaction with good left ventricular function and right ventricular hypertrabeculation

RV-EDVi: right ventricular end-diastolic volume index, RV-ESVi: right ventricular end-systolic volume index, RV-SVi: right ventricular stroke volume index, RV-EF: right ventricular ejection fraction, RV-TMi: right ventricular end-diastolic trabecular and papillary muscle mass index, RV-CMi: right ventricular end-diastolic compact myocardial mass index, RV-GLS: right ventricular global longitudinal strain, RV-FWS: right ventricular free-wall strain, RV-SS: right ventricular septal strain

The bold values indicate statistical significances (p<0.05).
